# Characterization of Divalent Metal Transporter 1 (DMT1) in *Brugia malayi* suggests an intestinal-associated pathway for iron absorption

**DOI:** 10.1016/j.ijpddr.2018.06.003

**Published:** 2018-06-20

**Authors:** Cristina Ballesteros, James F. Geary, Charles D. Mackenzie, Timothy G. Geary

**Affiliations:** a Institute of Parasitology, McGill University, 21111 Lakeshore Road, Sainte-Anne-de-Bellevue, Quebec, H9X 3V9, Canada; b Department of Biological Sciences, University of Notre Dame, Notre Dame, IN, USA; c Department of Pathobiology and Diagnostic Investigation, College of Veterinary Medicine, Michigan State University, East Lansing, MI, 48824, USA

**Keywords:** Lymphatic filariasis, Onchocerciasis, *Brugia malayi*, DMT1, Iron, *Wolbachia*

## Abstract

Lymphatic filariasis and onchocerciasis are neglected parasitic diseases which pose a threat to public health in tropical and sub-tropical regions. Strategies for control and elimination of these diseases by mass drug administration (MDA) campaigns are designed to reduce symptoms of onchocerciasis and transmission of both parasites to eventually eliminate the burden on public health. Drugs used for MDA are predominantly microfilaricidal, and prolonged rounds of treatment are required for eradication. Understanding parasite biology is crucial to unravelling the complex processes involved in host-parasite interactions, disease transmission, parasite immune evasion, and the emergence of drug resistance. In nematode biology, large gaps still exist in our understanding of iron metabolism, iron-dependent processes and their regulation. The acquisition of iron from the host is a crucial determinant of the success of a parasitic infection. Here we identify a filarial ortholog of Divalent Metal Transporter 1 (DMT1), a member of a highly conserved family of NRAMP proteins that play an essential role in the transport of ferrous iron in many species. We cloned and expressed the *B. malayi* NRAMP ortholog in the iron-deficient *fet3fet4* strain of *Saccharomyces cerevisiae*, performed qPCR to estimate stage-specific expression, and localized expression of this gene by immunohistochemistry. Results from functional iron uptake assays showed that expression of this gene in the iron transport-deficient yeast strain significantly rescued growth in low-iron medium. DMT1 was highly expressed in adult female and male *B. malayi* and *Onchocerca volvulus*. Immunolocalization revealed that DMT1 is expressed in the intestinal brush border, lateral chords, and reproductive tissues of males and females, areas also inhabited by *Wolbachia.* We hypothesize based on our results that DMT1 in *B. malayi* functions as an iron transporter. The presence of this transporter in the intestine supports the hypothesis that iron acquisition by adult females requires oral ingestion and suggests that the intestine plays a functional role in at least some aspects of nutrient uptake.

## Highlights

• BmDMT1 is a functional iron transporter.
• Expression of BmDMT1 rescues growth in an iron transport-deficient strain of yeast in low-iron medium.
• BmDMT1 is expressed in *B. malayi* microfilariae, L3, L4, adult females and adult males.
• BmDMT1 is highly expressed in the intestine, lateral cords and reproductive tissue in *B. malayi*.

## Graphical abstract

**Figure:**
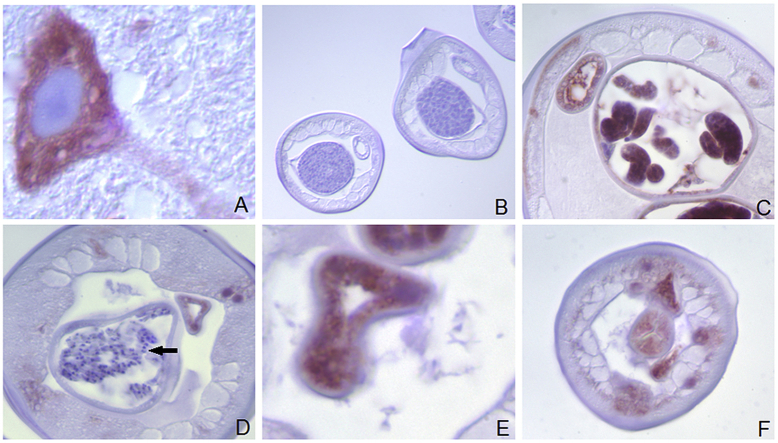

## 1 Introduction

The human filarial diseases lymphatic filariasis (LF) and onchocerciasis affect around 150 million people in tropical and subtropical regions. LF is a debilitating disease caused by infection with the filarial nematodes *Wuchereria bancrofti*, *Brugia malayi*, and *Brugia timori* and is endemic in 60 countries, affecting approximately 120 million people with 1.23 billion people at risk of infection (WHO, 2014b). Onchocerciasis (river blindness), caused by *Onchocerca volvulus*, is endemic in 24 countries, almost all in sub-Saharan Africa, with close to 25 million people infected (WHO, 2014a; 2014b). Control and elimination programmes primarily through mass drug administration (MDA), with vector control where appropriate, aim to reduce transmission of these neglected tropical diseases and to alleviate suffering and disability (APOC, 2005; Ottesen, 2000). Although most filarial species that parasitize humans have no convenient laboratory animal host, *B. malayi* can readily infect a variety of rodents and has served as an important model organism for filarial nematodes in the research community (Williams et al., 2000).

Although MDA has markedly reduced transmission of human filarial parasites, the drugs used in control programmes are predominantly microfilaricidal and do not kill the adult worms, which can remain in the host for many years. Thus, repeated rounds of MDA are required to lower transmission rates and eventually break the infection cycle, engendering a risk of potential emerging resistance to these drugs. Although simultaneous administration of albendazole + ivermectin + diethylcarbamazine appears to be macrofilaricidal against *W. bancrofti* (Thomsen et al., 2016; Fischer et al., 2017), this finding needs to be confirmed. Similar results may be attainable in onchocerciasis, but the use of diethylcarbamazine in onchocerciasis regions poses significant concerns of risk. It is prudent, therefore, to continue the search for selectively macrofilaricidal drugs.

Recent transcriptomic and proteomic studies of nematodes have greatly enhanced our knowledge in this area; however, many gaps remain. Filling the gaps could help us further understand how parasites interact and thrive within their hosts, how they evade the immune system and how drug resistance emerges. An area of increasing interest is iron and heme biology. The host-pathogen interplay over the control of iron homeostasis influences the course of infectious diseases to favor the host or the pathogen (Nairz et al., 2010; Payne, 1993; Wessling-Resnick, 2015). Iron is an essential trace element required for many metabolic pathways that require hemoprotein functions. However, iron is pro-oxidant and is present at very low free concentrations in biofluids. Complex regulatory mechanisms are necessary for adequate absorption, trafficking, utilization, storage and elimination of iron (Wheby et al., 1964). Because iron is so important yet so tightly regulated and unavailable, pathogens have evolved sophisticated ways of acquiring iron from the host, including the expression of receptors that can bind iron transport proteins such as transferrin, lactoferrin or hemoglobin, or by the use of low molecular weight iron chelators known as siderophores (Glanfield et al., 2007; Sutak et al., 2008; Tachezy et al., 1996; Weinberg, 2009; Wilson et al., 1994). A long-standing question pertaining to filariae has been, “How do filarial worms obtain nutrients?”. Is the gut functional or do filariae acquire nutrients solely across the cuticle? Proteomic evidence suggests that the gut is functional, as the intestine is rich in proteolytic enzymes and transporters that may be involved in absorption and digestion of nutrients (Morris et al., 2015). A recent study has also shown that a heme transporter (BmHRG-1) is functional in *B. malayi* and localizes both to the endocytic compartments and cell membrane when expressed in yeast (Luck et al., 2016), further supporting the concept that the gut plays a role in nutrient acquisition.

In *B. malayi* and many other filariid species, the obligate endosymbiotic bacteria *Wolbachia* encodes the heme biosynthetic genes required for the survival of its host parasite, making this pathway an attractive anti-filarial drug target (Strubing et al., 2010; Wu et al., 2009). However, the parasite must acquire iron for heme biosynthesis from its host, a process that is also crucial for its survival. In mammals, divalent metal transporter 1 (DMT1), a protein belonging to the highly conserved NRAMP (natural resistance-associated macrophage proteins) family (Cellier et al., 1995), plays an essential role in the transport of ferrous iron across the brush border of the intestine (Han et al., 1999). Interestingly, DMT1 is also highly expressed in neurons and plays a role in iron transport at the blood brain barrier (Skjorringe et al., 2015), and is also expressed in human placenta where it may play a role in the release of iron from endosomes to the cytoplasm in placental syncytiotrophoblasts (Li et al., 2012).

We hypothesized that a homolog of DMT1 in *B. malayi* plays a role in iron transport and that it is expressed in the parasite intestine. We identified a NRAMP-like transporter (XM_001899422.1) in the *B. malayi* genome that is highly similar to human DMT1. A cDNA encoding this protein was cloned and expressed in an iron-deficient *fet3fet4* strain of *Saccharomyces cerevisiae*, characterized its stage-specific expression, and used immunohistochemistry to localize expression of this gene.

## 2 Materials and methods

### 2.1 Identification of *Brugia malayi* DMT1 and protein sequence characteristics

NRAMP sequences from several species were blasted against the *B. malayi* genome in GenBank™ using the National Center for Biotechnology Information (NCBI) Blast program. We identified an apparent *B. malayi* NRAMP sequence (XM_001899422.1) and cloned a cDNA encoding it for functional studies in yeast. The encoded amino acid sequence was aligned to several NRAMP amino acid sequences using PRALINE (http://www.ibi.vu.nl/programs/pralinewww/) (Simossis and Heringa, 2003, Fig. 1). To view predicted transmembrane domains, we used the web-based software Protter (http://wlab.ethz.ch/protter; Ottesen, 2000) which gathers protein features from various annotation sources such as Uniprot. A topology map of *B. malayi* DMT1 is shown in Fig. 2. The Expasy (Expert Protein Analysis System) ProtParam (Wilkins et al., 1999) tool was used to obtain characteristics such as predicted amino acid sequence and molecular weight.

**Fig. 1:**
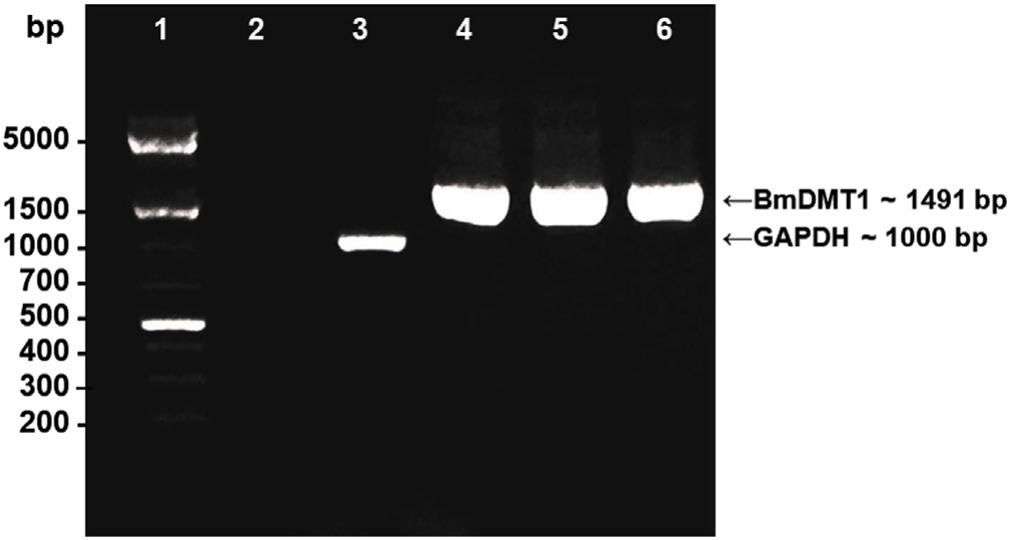
PCR amplification of BmDMT1. BmDMT1 cDNA was amplified by PCR from adult female *B. malayi* total RNA and products separated by electrophoresis through a 1% agarose gel and stained with ethidium bromide. Lane 1: O'GeneRuler 1 Kb Plus DNA Ladder (Thermo Fisher Scientific Inc., USA); Lane 2: minus-reverse transcriptase (-RT) control; Lane 3: GAPDH endogeneous control corresponding to a size of about 1000 bp; Lanes 4–6: BmDMT1 technical replicate PCR products corresponding to a predicted size of 1491 bp.

**Fig. 2:**
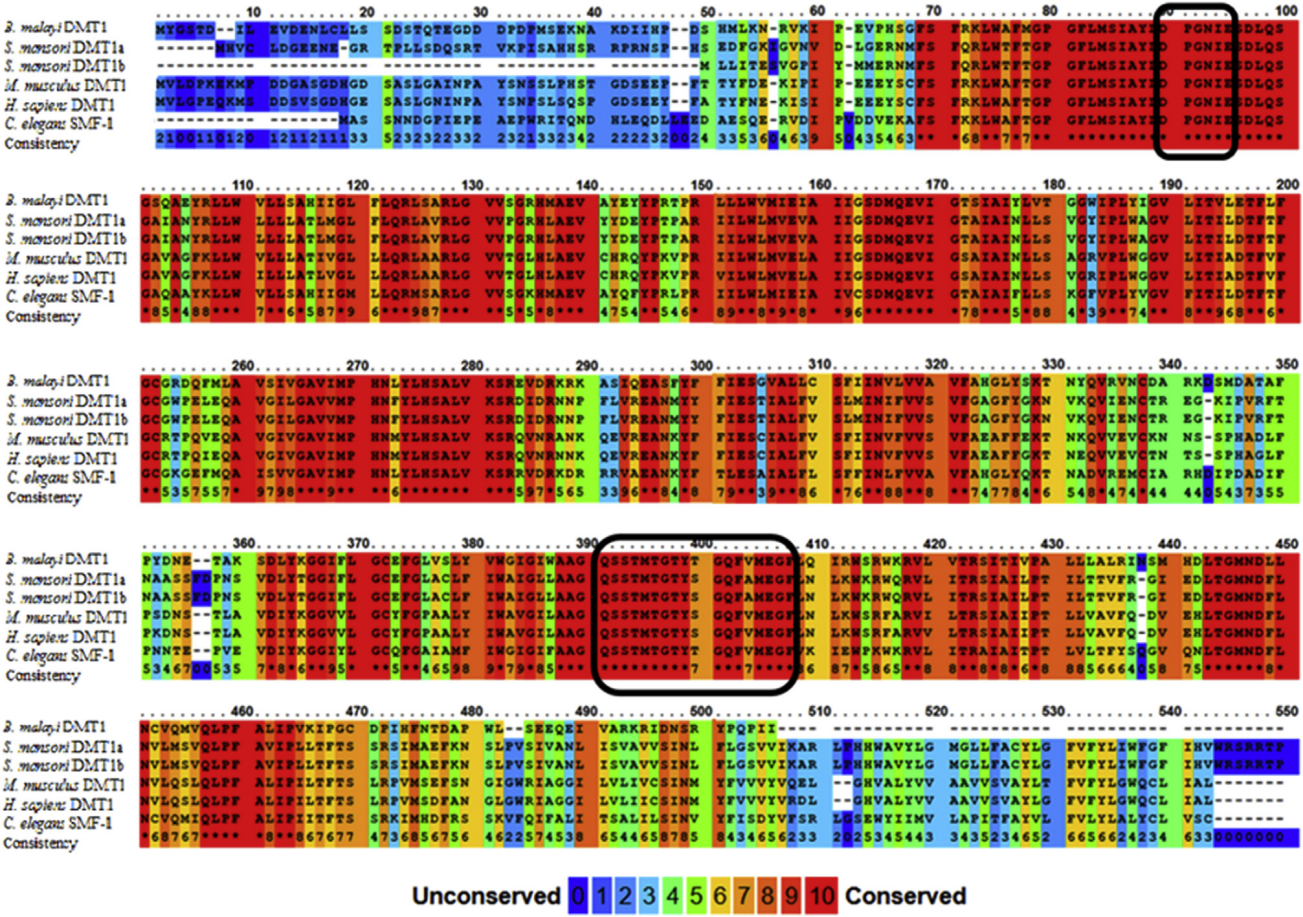
Multiple sequence alignment of BmDMT1 with NRAMP sequences from various species. The alignment was made using PRALINE (Simossis and Heringa, 2003). The scoring scheme designates 0 for the least conserved alignment position, shaded in dark blue, to 10 for the most conserved alignment position, shaded in red. The conserved consensus transport sequence involved in divalent metal ion translocation and the conserved DPGN motif important for metal ion binding are boxed in black. (For interpretation of the references to colour in this figure legend, the reader is referred to the Web version of this article.)

### 2.2 cDNA synthesis, polymerase chain reactions and cloning of BmDMT1

Total RNA from adult female *B. malayi* was obtained from the Molecular Resources Division of the NIH-NIAID Filariasis Research Reagent Research Center (Smith College, Northampton, MA). RNA was subjected to cDNA synthesis using SuperScript III reverse transcriptase (Invitrogen, 18080) using the manufacturer's protocol. PCR amplification of the *B. malayi* NRAMP-like transporter mRNA (Genbank accession: XM_001899422.1) was performed using the forward primer 5′-GCCATAAAGCTTATGTATGGAAGTACTGATATACTTG-3’ (*Hin*dIII site underlined) and reverse primer 5′-GCCATATCTAGATTAGATGATAGGTTGAGGG -3’ (Xbal site underlined). Primers were designed using Geneious Pro (version 4.8.5) based on sequence information from NCBI (http://www.ncbi.nlm.nih.gov/gene). PCR was carried out using the Phusion High-Fidelity DNA Polymerase kit (Invitrogen Life Technologies, Burlington, ON) in a 50 μl reaction volume using 1X Phusion HF Buffer, 200 μM dNTPs, 0.5 μM each primer, 20 ng cDNA and 0.02 U/μl Phusion DNA polymerase. A gradient PCR reaction was performed on an Eppendorf Mastercycler (Eppendorf, Westbury, NY) for a total of 30 cycles: 10 s at 98 °C, 20 s at 52 °C with 1° increments every cycle and 30 s at 72 °C for 10 cycles followed by 10 s at 98 °C, 20 s at 65 °C and 30 s at 72 °C for 20 cycles. The PCR product was cloned into the pYES2 yeast expression vector (Invitrogen, Carlsbad, CA) for functional analysis studies and transformed into *Escherichia coli* DH5-α strain for propagation. pYES2 allows selection of transformants by uracil prototrophy and has the yeast GAL1 promoter for inducible protein expression by galactose and ampicillin resistance for selection in *E. coli*. A GeneJet Plasmid Mini-Prep kit (Thermo Fisher Scientific, Waltham, MA) was used to purify plasmids from DH5-α and cloning of the DMT1 sequence was confirmed by sequencing at the McGill University and Génome Québec Innovation Center (http://gqinnovationcenter.com).

### 2.3 Functional expression of BmDMT1 cDNAs in yeast

To study the function of *B. malayi* DMT1, we used the *Saccharomyces cerevisiae fet3fet4* double mutant (strain DEY1453), which is defective in low- and high-affinity iron transport systems (Eide, 1998) and grows poorly on iron-limited medium. The parent strain DY150 was used as a positive control. These strains were kindly provided by Dr. Jerry Kaplan (University of Utah, Salt Lake City, UT). DEY1453 and DY150 were transformed by electroporation with the *B. malayi* DMT1 sequence cloned into pYES2 or the empty pYES2 vector as a negative control. pYES2 contains the URA3 gene, which allows for the selection of transformants by growth on uracil-lacking media (Invitrogen, Carlsbad, CA). The protocol used for transformation was as described with slight modifications (Thompson et al., 1998). An overnight culture was diluted to OD = 0.4 and allowed to grow for several hr. Cells were pelleted by low-speed centrifugation at 4 °C for 5 min, washed twice with 25 ml ice-cold water and then washed in 10 ml ice-cold 1 M sorbitol. Cells were pelleted again and 500 μl sorbitol (1 M) was mixed with the pellet. Eighty μl of the cell suspension was mixed with 1–3 μg plasmid DNA in a 2 ml Eppendorf tube, incubated on ice for 5 min and then transferred to a 0.2 cm electroporation cuvette. Cells were pulsed once at 1.5 kV, 25 uFD, 200 ohms (Bio-Rad Gene Pulser) and then immediately 1 ml ice-cold sorbitol (1 M) was added. Electroporated cells were then incubated at 30 °C (without shaking) for 2 hr. After 2 hr, 150–200 μl of the cell suspension was plated and incubated at 30 °C for several days on URA^−^ selective synthetic defined (SD) media supplemented with adenine hemisulfate and 20% glucose until colonies formed.

Overnight cultures were prepared by inoculating a single colony in selective SD URA-liquid culture media at 30 °C with shaking. Overnight cultures were diluted to OD_600_ = 1.0 and serial dilutions prepared (1.0, 0.1, 0.01, and 0.001). Five μl of each dilution was spotted on a selective SD medium URA^−^ plate supplemented with adenine hemisulfate, 20% galactose to induce expression of the DMT1 insert, 10% raffinose and either 2 or 20 μM ferric chloride or 100 μM or 200 μM ferrous ammonium sulfate. Plates were incubated at 30 °C until colonies formed.

### 2.4 Stage-specific expression of BmDMT1

Stage-specific expression of *B. malayi* DMT1 was determined by quantitative polymerase-chain reaction (qPCR) in microfilariae, L3, L4, adult females and adult males. Total RNA for these stages was obtained from the Molecular Resources Division of the NIH-NIAID Filariasis Research Reagent Research Center (Smith College). Briefly, 100 ng of the original total RNA sample were reverse transcribed (SuperScript VILO MasterMix, Invitrogen) and diluted five-fold for subsequent qPCR amplifications. For each sample, assays were carried out in quadruplicate in 20 μl-reaction volumes containing 10 μl 2X SYBR Select Master Mix (Thermo Fisher Scientific), 200 nM final concentration each forward and reverse primer, and 2 μl cDNA. Amplifications were run in an ABI 7500 real-time PCR system using the following program: 50 °C for 2 min, 95 °C for 2 min, 40 cycles of 95 °C for 15 sec, 58 °C for 15 sec, 72 °C for 1 min, followed by a melt curve. No-template controls were run to verify the absence of genomic DNA. Specific primers were designed in Primer-BLAST (http://www.ncbi.nlm.nih.gov/tools/primer-blast/) (Ye et al., 2012) for *B. malayi* DMT1 and *B. malayi* glyceraldehyde-3-phosphate dehydrogenase (GAPDH), which was used as a normalizer. The oligonucleotides used to amplify DMT1 were: forward 5′-GCCTTTATGGGTCCCGGATT-3′ and reverse 5′-CAGCTTGTGACCCTGATTGC-3’. For GAPDH, the oligonucleotides used were: forward 5′- TTTCTGCAGAGGGAGGCAAG-3′ and reverse 5′- TCAGCGGGATCTTTGCTGTT-3’. PCR efficiency curves for each gene were generated using four duplicate 5-fold dilutions of cDNA. Relative expression was calculated using the ΔΔCt method (Livak and Schmittgen, 2001) for relative quantification of each gene normalized to GAPDH relative to expression in adult females.

### 2.5 Western blot analysis of BmDMT1 with a commercial polyclonal antibody

*B. malayi* DMT1 has high homology with human NRAMP 2 isoform 3 [GenBank: NP_001167599.1], with 61% identity and 74% similarity, indicating a high likelihood that an antibody raised against the human protein would recognize the *B. malayi* protein. A rabbit polyclonal antibody raised against a peptide derived from human DMT1 (Abcam, Cat: ab123085, Toronto, ON), corresponding to amino acids 261–291, was tested. This region in the *B. malayi* protein is 71% identical to the human DMT1 sequence.

To extract crude protein, adult *B. malayi* females obtained from the Filariasis Research Reagent Repository Center (Athens, GA), were pooled and rinsed in sterile phosphate-buffered saline (PBS), transferred to a 1.5 ml microcentrifuge tube (VWR International, Mississauga, ON) and suspended in PBS. Worms were mechanically broken by freezing/thawing in liquid N_2_ five times, crushing with a glass pipette and homogenizing by needle passage (from 23G to 30G). This was followed by sonication using a Branson digital sonifier on ice 3 times for 5-sec pulses with 5-sec intervals. Soluble material was extracted into PBS during an overnight incubation at 4 °C on a rotator followed by centrifugation at 12172*g* for 60 min at 4 °C. The supernatant (soluble extract) was transferred to a new 1.5 ml Eppendorf tube and stored at −20 °C. To extract membrane protein, the pellet was resuspended in PBS containing 0.5% Triton X-100 and SigmaFAST™ Protease Inhibitor Cocktail (Sigma-Aldrich, St. Louis, MO), homogenized by needle passage as before, incubated for 3 hr at 4 °C on a rotator and then centrifuged at 46000 g for 60 min at 4 °C. The supernatant (TX extract) was transferred to a 1.5 ml Eppendorf tube and stored at −20 °C until further use. Protein concentrations of soluble and membrane protein extracts were determined by the Bradford method (Pierce, Rockford, IL).

For Western blot analysis, 20 μg soluble and membrane proteins were first separated on a 4–15% gradient polyacrylamide gel (Mini-Protean TGX Precast Protein Gels, Bio-Rad, Hercules, CA) by electrophoresis for 1.5 hr at 125 V. Proteins were transferred to a polyvinylidene difluoride (PVDF) membrane overnight at 10 mA. The membrane was blocked with 5% w/v nonfat dry milk for 1 hr, washed 3 times for 5 min in Tris-buffered saline-Tween 20 (TBS-T) and then incubated with the primary anti-DMT1 antibody (Abcam, Cat: ab123085) diluted at 1:500 in 1% w/v nonfat dry milk overnight at 4 °C. After incubation with primary antibody, the PVDF membrane was washed 3 times for 5 min in TBS-T and incubated with enhanced chemiluminescent (ECL) peroxidase labeled anti-rabbit secondary antibody (1:5000) (GE Healthcare, Mississauga, ON) for 1 hr at room temperature. The membrane was washed 3 times in TBS-3T for 5 min and then another 3 times in TBS-T. Antibody binding was visualized by SuperSignal West Femto maximum sensitivity substrate (Thermo Fisher Scientific) according to the manufacturer's protocol on a MyECL Imager (Thermo Fisher Scientific).

A negative control cell lysate (HEK 293) and a positive control cell lysate (Caco-2) were also probed with the anti-DMT1 antibody and a rabbit polyclonal anti-GAPDH antibody (Proteintech, Rosemont, IL, Cat: 10494-1-AP) was used as an endogenous control for *B. malayi*, HEK293 and Caco-2 cell lysates.

### 2.6 Histological preparation and immunolocalization of BmDMT1

An immunohistochemistry approach was used to localize the expression of *B. malayi* DMT1. Specimens were fixed in 10% neutral buffered formalin then placed in HistoGel™ (Richard-Allan Scientific, Kalamazoo, MI). This was followed by processing, embedding in paraffin and sectioning on a rotary microtome at 4–5 μm. Slides were deparaffinized in xylene and hydrated through descending grades of ethyl alcohol to distilled water.

*O. volvulus* nodules were collected as part of pre-ivermectin nodulectomy campaigns in Ecuador and Cameroon from individuals who were known not to have received antifilarial treatment. Nodules were processed, embedded in paraffin and sectioned on a rotary microtome at 4 μm as described above. Sections were placed on slides coated with 3- aminopropyltriethoxysilane and dried at 56 °C overnight. The slides were subsequently deparaffinized in xylene and hydrated through descending grades of ethyl alcohol to distilled water.

Following pretreatment, standard avidin-biotin complex staining steps were performed at room temperature on a DAKO Autostainer. Nonspecific protein binding was blocked with normal goat serum (Vector Labs, Burlingame, CA) for 30 min and sections were then incubated with an avidin/biotin blocking system for 15 min (avidin D – Vector Labs/d-biotin – (Sigma Aldrich)). Primary antibody slides were incubated for 60 min with polyclonal rabbit anti-DMT1 antibody (Abcam, ab123085; synthetic peptide conjugated to KLH, corresponding to amino acids 261–291 of human DMT1) diluted 1:100 in Normal Antibody Diluent (NAD) (Scytek, West Logan, UT). Slides were incubated for 30 min in biotinylated goat anti-rabbit IgG (H + L) prepared at 11.0 μg/ml in NAD followed by a 30 min incubation in R.T.U. Vectastain Elite ABC Reagent (Vector Laboratories). Vector Nova Red peroxidase chromogen (Vector Laboratories) was used to develop the reaction for 15 min followed by 15 min counterstain in Gill Hematoxylin (Thermo Fisher Scientific), differentiation, dehydration, clearing and mounting with synthetic mounting media.

Sections of mouse brain (WT C57Bl6 and Balb/c mice) prepared by the histological procedures described above were used as controls, as DMT1 is known to be present in CNS neuronal bodies.

## 3 Results

### 3.1 *B. malayi* DMT1 primary sequence analysis

Molecular cloning of the *B. malayi* NRAMP-like transporter (Genbank sequence: XM_001899422.1) generated a 1491 bp cDNA encoding an open reading frame of 496 amino acids (XP_001899457_1) with a predicted molecular mass of 55,774 Da. This region was amplifed from total RNA and expressed for functional studies in yeast. As this sequence was based on an earlier version of the *B. malayi* genome (Assembly ASM299v2), a BLASTN was later performed with this sequence against the current *B. malayi* genome version (*B.malayi*-4.0) in Wormbase Parasite (http://parasite.wormbase.org), which revealed an overlap with gene Bm13827. Comparison of these coding sequences by multiple sequence alignment showed that the first 1371 bases and the last 120 bases are identical between them, with an internal region consisting of 261 bases absent from the XM_001899422.1 sequence. Further analysis of the Bm13827 sequence revealed splice sites at the 5′ and 3′ end of these 261 bases. For future reference in this paper, we term the encoded protein: BmDMT1. That this sequence was obtained via PCR with 5′ and 3′ end primers indicates that it is expressed in the parasite. Gel electrophoresis revealed that these primers amplified only one PCR product from total RNA (Fig. 1).

### 3.2 Multiple sequence and secondary protein sequence analysis of BmDMT1

BmDMT1 was compared to other sequences in the Genbank database (Fig. 2) by multiple sequence alignment. BmDMT1 has high identity to sequences in the NRAMP family of proteins in *C. elegans* (62%), DMT1A and DMT1B in *Schistosoma mansoni* (63 and 62%, respectively), *Drosophila malanogaster* (61%), mouse (61%), human (61%), *Xenopus tropicalis* (60%) and yeast (29%). The sequence also contains a conserved consensus transport sequence involved in divalent metal ion translocation and a conserved DPGN motif important for metal ion binding. This DPGN region contains conserved residues which can coordinate Mn^2+^, Fe^2+^ and Cd^2+^, but not Ca^2+^, and is in the first extracelluar loop of the DMT1 sequence (Wu et al., 2013).

**Fig. 3:**
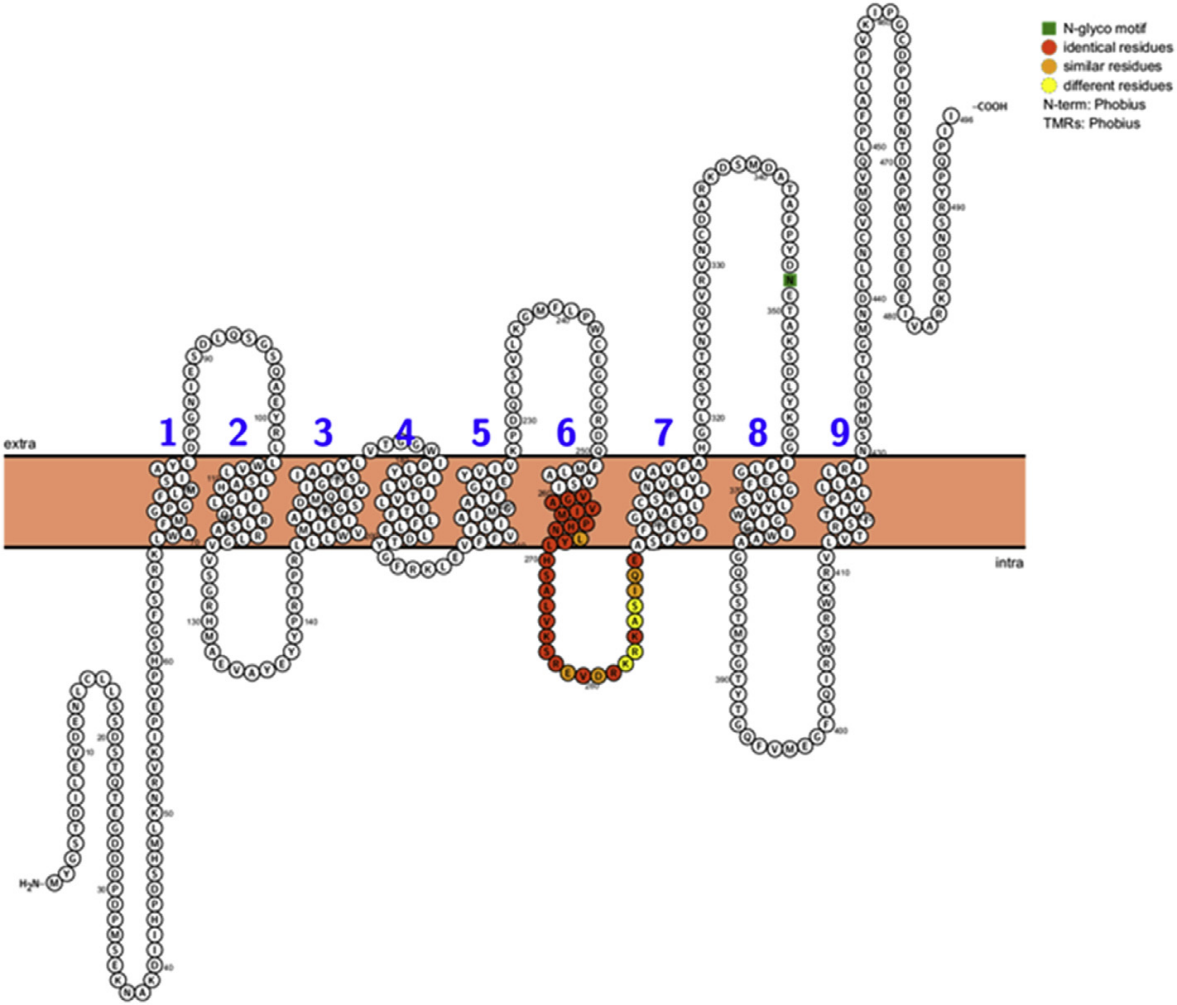
Predicted transmembrane topology map of BmDMT1. The topology map was generated using the Protter server (Ottesen, 2000) and shows nine transmembrane regions and one *N*-glycosylation motif at amino acid position 348 (in green). The region corresponding to amino acids 261–291 of human DMT1 (NP_001167599.1) targeted by the anti-DMT1 antibody (Abcam, ab123085) is shown with identical, similar, and different residues highlighted. This region is 71% identical and 87% similar to human DMT1. (For interpretation of the references to colour in this figure legend, the reader is referred to the Web version of this article.)

Sequence comparison and a predicted topology map of transmembrane domains shows nine transmembrane regions and one *N*-glycosylation motif at amino acid position 348, an intracellular N-terminal end and an extracellular C-terminal end (Fig. 3).

### 3.3 Functional analysis of BmDMT1 in yeast

**Fig. 4:**
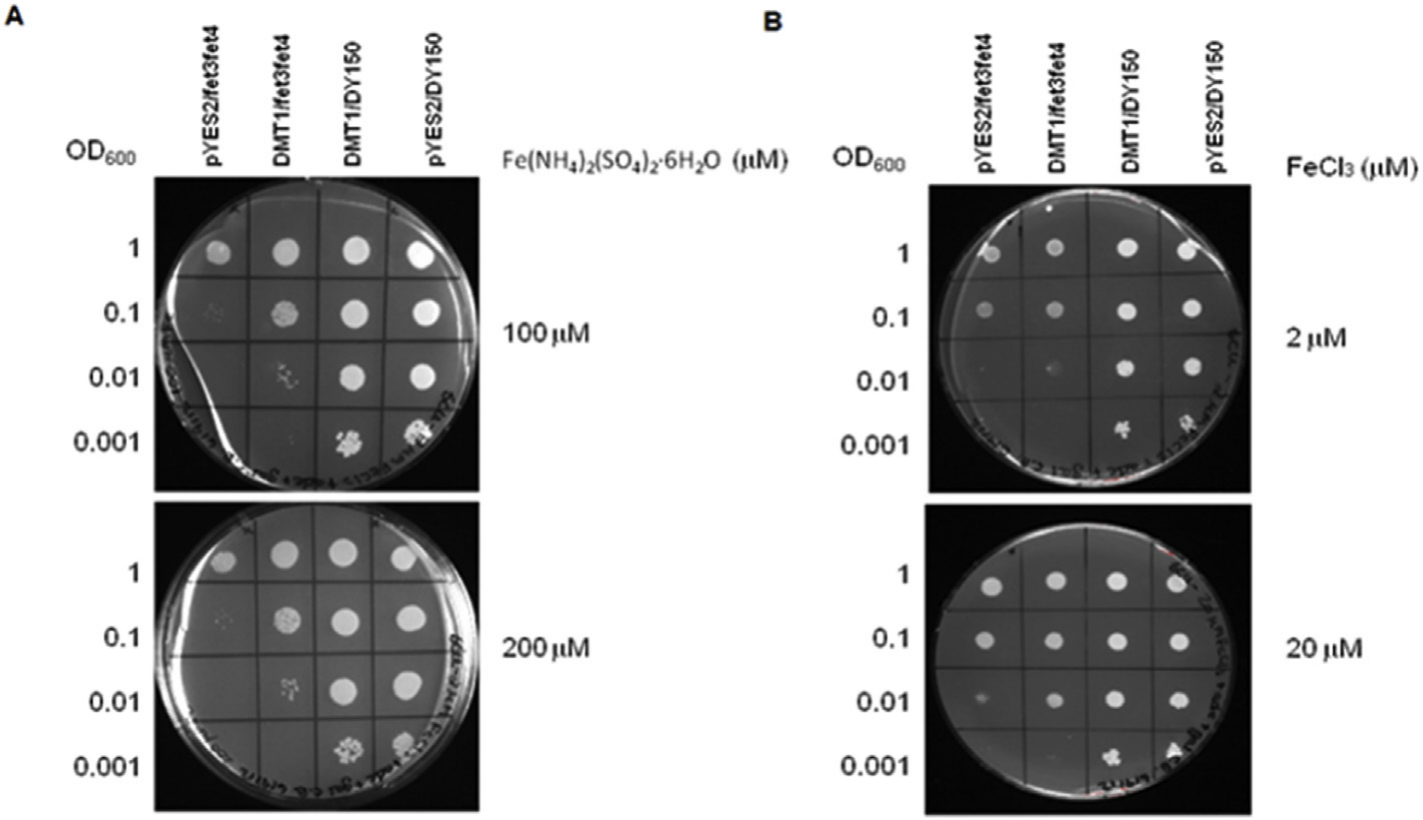
Functional analysis of BmDMT1 in yeast. The *fet3fet4* mutant yeast strain was transformed with BmDMT1 or the empty PYES2 expression vector. The DY150 parent strain was used as a positive control and transformed with either BmDMT1 or the empty vector. Serially diluted cells were spotted on URA-selective plates supplemented with either A. 100 μM or 200 μM ferrous ammonium sulfate, or B. 2 μM or 20 μM ferric chloride.

To test the ability of BmDMT1 to transport ferrous iron, a cDNA sequence (XM_001899422.1) encoding it was amplified from total RNA, sequenced and cloned into the pYES2 yeast expression vector and transformed into the *fet3fet4* iron transport deficient strain of *S. cerevisiae*. This strain grows poorly on low iron plates because the high (*fet3*) and low (*fet4*) affinity Fe^2+^ transporters have been mutated (Eide et al., 1992). Expression of this sequence significantly rescued the growth phenotype in this strain (Fig. 4A and Fig. 4B) on media supplemented with either 2 or 20 μM ferric chloride, or 100 or 200 μM ferrous ammonium sulfate.

### 3.4 Stage-specific expression of BmDMT1

**Fig. 5:**
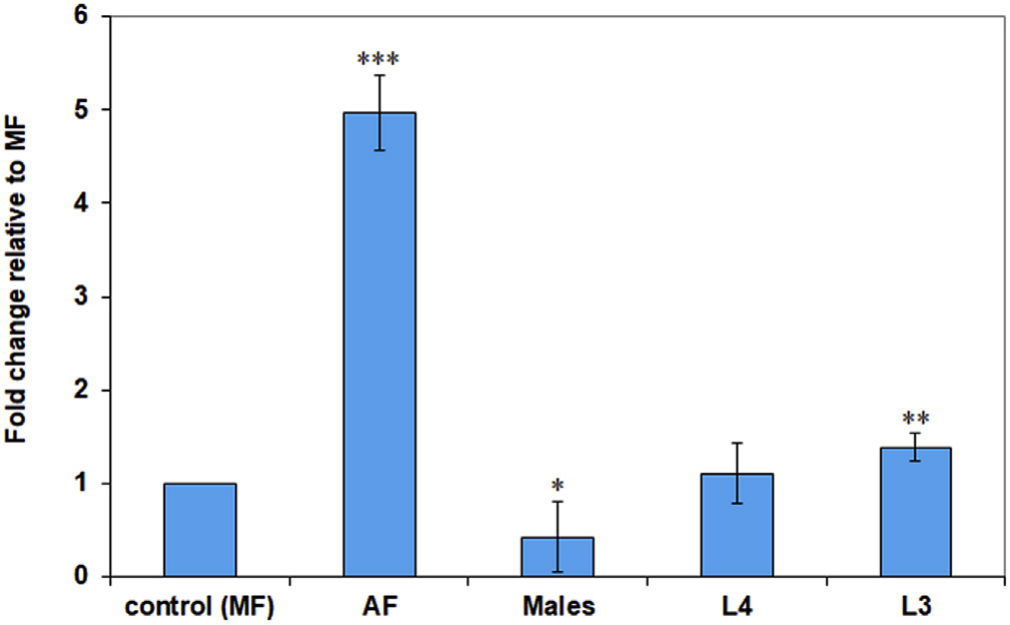
Relative BmDMT1 Expression levels in different *B. malayi* life stages. Fold changes were calculated using the ΔΔCT method using GAPDH as a normalizer and relative to expression levels in microfilariae. *, **, *** p-values, < 0.05, <0.01, <0.001, respectively, according to unpaired *t*-test.

Expression of BmDMT1, estimated by quantitative PCR amplification, was detected in microfilariae, L3, L4, adult females, and adult males. The ΔΔCt method, normalized to GAPDH, was employed to determine fold change (FC) relative to expression in MF. Mean CT values and calculated fold changes relative to MF are shown in Table 1 and fold changes plotted in Fig. 5. Primer amplification efficiencies were determined using four duplicate 5-fold dilutions of cDNA and were 91% for GAPDH (normalizer) and 94% for BmDMT1.

**Table 1:** Fold change expression of BmDMT1 relative to MF, calculated by ΔΔCT method.

Sample	Average^a^ CT GAPDH	Average^a^ CT BmDMT1	ΔCT (BmDMT1-GAPDH)	ΔΔCT	Fold Difference in BmDMT1 relative to MF
MF (Control)	21.20 ± 0.08	25.77 ± 0.09	4.57 ± 0.12	0.00 ± 0.12	1 (0.92–1.09)
AF	27.96 ± 0.02	30.20 ± 0.15	2.24 ± 0.15	−2.33 ± 0.15	5.06 (4.53–5.58)
L4	24.60 ± 0.20	29.15 ± 0.09	4.56 ± 0.21	−0.01 ± 0.21	1.02 (0.87–1.16)
L3	21.54 ± 0.14	25.64 ± 0.21	4.10 ± 0.25	−0.47 ± 0.25	1.41 (1.16–1.65)
M	21.04 ± 0.05	26.43 ± 0.09	5.39 ± 0.10	0.82 ± 0.10	0.57 0.53–0.61

a Average of 4 replicates.

### 3.5 Western blot analysis of BmDMT1 with a commercial polyclonal antibody

**Fig. 6:**
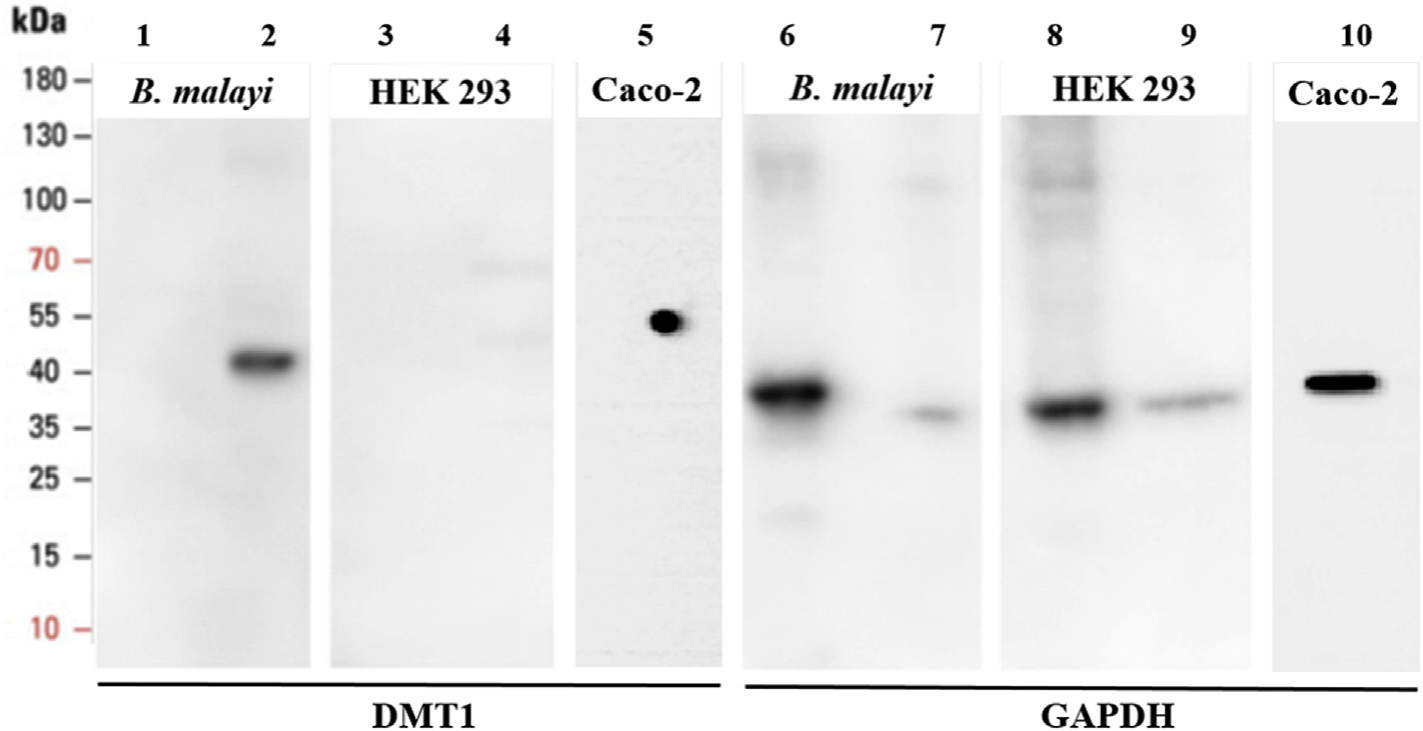
Western blot detection of divalent metal transporter 1 (DMT1) in *Brugia malayi* adult females. Soluble and membrane protein was extracted from *B. malayi* adult females (soluble protein lanes 1 and 6; membrane protein lanes 2 and 7) and HEK293 cells (soluble protein lanes 3 and 8; membrane protein lanes 4 and 9). In addition, a whole cell protein lysate was extracted from human Caco-2 cells (lanes 5 and 10). Approximately 20 μg of protein from each extract was subjected to SDS-PAGE followed by Western blot analysis with rabbit polyclonal anti-DMT1 antibody (Abcam, Cat: ab123085) (lanes 1 to 4) and rabbit polyclonal anti-GAPDH antibody (Proteintech, Cat: 10494-1-AP) (lanes 5 to 8).

*B. malayi* soluble and membrane proteins were probed with a rabbit polyclonal antibody to human DMT1. Western blot results showed a single band with an apparent molecular weight of 43 kDa (Fig. 6), lower than the predicted band of 55.7 kDa. Due to their hydrophobic nature, membrane proteins can associate with a larger number of SDS molecules than soluble proteins of the same molecular mass. For this reason, membrane proteins tend to migrate faster through SDS-PAGE and therefore their molecular mass can appear lower than actual values (Abeyrathne et al., 2010). As expected, we did not detect a DMT1 band in the soluble protein fraction. No DMT1 band was detected in the negative control HEK293 cell lysates and a single band at an expected 55 kDA was detected in the human Caco-2 cell lysate; a band at approximately 37 kDa (molecular mass of GAPDH) was detected mainly in the soluble protein extracts for both *B. malayi* and HEK293 cells and a fainter band in the membrane extract. A band was also detected at approximately 37 kDA in human Caco-2 cell lysate.

### 3.6 Immunolocalization of BmDMT1

**Fig. 7:**
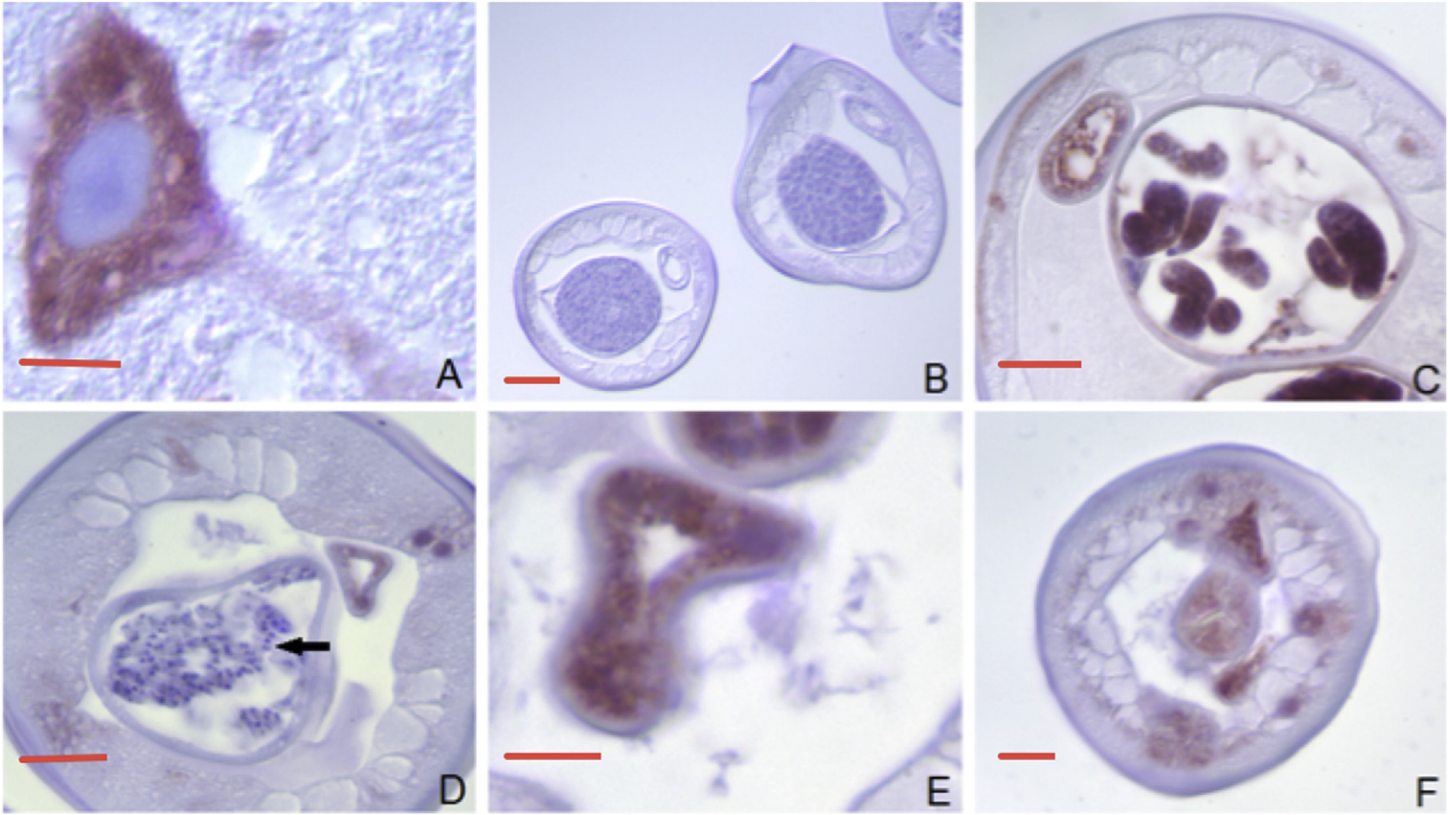
DMT1 immunocytochemical staining in control tissues and *B. malayi.* A. Mouse CNS neuron staining positively for DMT1 (positive control) (bar = 5 μ). B. Negative control male *B. malayi* (without primary antibody) (bar = 70 μ). C. Uterine “sausage forms” are positive; the gut wall and the hypodermis are also positive (bar = 50 μ). D. Male worm showing spermatozoa (arrow) free of staining (Bar = 50μ). E. Strongly staining gut wall of a male worm (bar = 20 μ). F. Anterior end of a male worm: The wall of the pharynx and the hypodermis are moderately to strongly positive (bar = 50 μ).

**Fig. 8:**
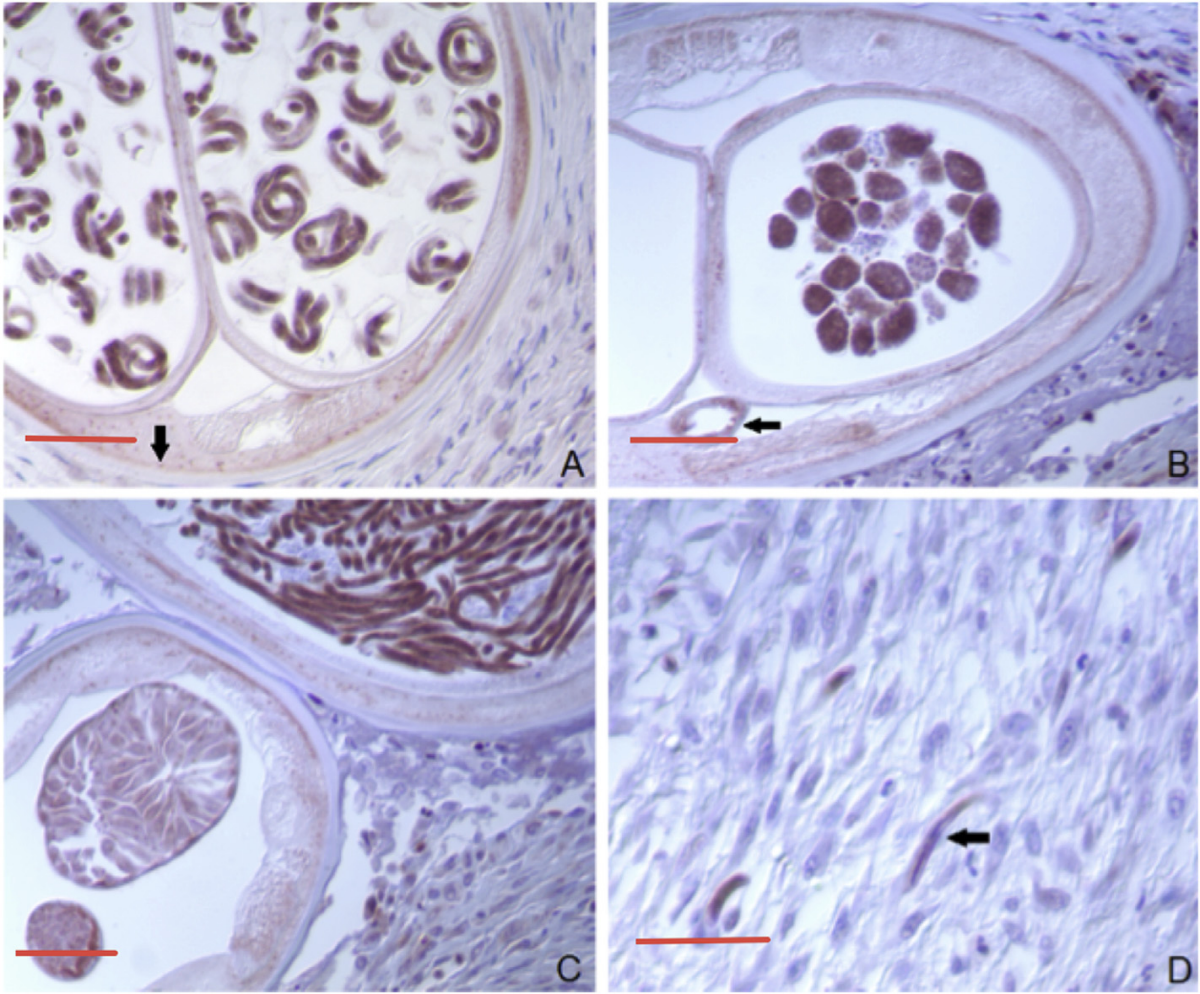
DMT1 immunocytochemical staining in *O. volvulus*. A. Microfilariae within egg shells are variably positive; the hypodermis (arrow) is also moderately positive (bar = 90 μ). B. Well-developed morulae are positive, while those under-developed or in transition are less positive. The gut wall (arrow) also is positive (bar = 80 μ). C. Stretched microfilariae are strongly positive (top right), early developing oocytes are lightly positive and the areas of the wall of the reproductive tract are strongly positive (bar = 70 μ). D. Emerged microfilariae in the outer areas of onchocercal nodules are DMT1 positive (arrow) (bar = 30 μ).

Immunohistochemistry of sections of adult male and female *B. malayi* (Fig. 7) and *O. volvulus* females in nodules (Fig. 8) with a rabbit polyclonal antibody to human DMT1 showed antibody binding in the intestinal region and lateral chords of males and females as well as in developing reproductive structures in females and spermatocytes in males. In the female reproductive tissues of both *B. malayi* and *O. volvulus*, intense staining was seen in uterine morulae. In *O. volvulus,* intense staining was also observed in the sausage form of microfilariae. Table 2 shows the intensity scoring for anti-DMT1 staining for different locations in *O. volvulus* and *B. malayi.*

**Table 2:** DMT1 location and intensity scoring.

	Species	Location	IC Intensity^a^	IC Intensity
Controls	Mouse	CNS Neurons	+++	
		Basal area gut epithelium	++	

Nematodes	Filarial Parasites		*O. volvulus*	*B. malayi*
		Cuticle	–	–
		Hypodermis	+ to ++	+ to ++
		Body wall muscle	– to +	–
		Intestinal wall	+	++
		Uterine wall	++	+ to ++
		Uterine oocytes	–	–
		Uterine morulae	++	+ to +++
		Uterine microfilariae	– to +++	– to +
		Emerged microfilariae	+ to +++	
		Intra-male spermatozoa	–	–

a IC Intensity level: Based on the overall intensity observed in all positive locations compared to tissue control in the same section. Range: no staining (−); weak staining (+); moderate staining (++); strong staining (+++).

## 4 Discussion

Iron is a crucial trace element as a component of hemoproteins that play essential roles in energy metabolism, cell growth, replication, respiration and DNA synthesis. However, free iron is extremely toxic and can generate free radicals which can damage cell membranes and DNA (Ponka, 1999). Hence, the storage and transport of iron are tightly regulated. In the diet of animals, iron can be found either sequestered in heme or in various non-heme forms. NRAMP proteins such as DMT1 are a family of highly conserved hydrophobic integral membrane proteins containing 10 to 12 transmembrane domains, depending on the species (Cellier et al., 1995), that play a role in transporting ferrous iron and other transition metal ions across membranes (Garrick et al., 2003; Mackenzie et al., 2006). In humans and other mammals, DMT1 is mainly expressed in the intestinal brush border, where it has a crucial function in ferrous iron absorption and iron utilization by erythroid cells (Canonne-Hergaux et al., 2001). DMT1 also has a role in iron transport at the blood-brain barrier (Skjorringe et al., 2015) and is present in the outer mitochondrial membrane (Wolff et al., 2014). Iron is an essential cofactor in hemoproteins required for energy metabolism in mitochondria, so the presence of DMT1 in the outer mitochondrial membrane is not surprising.

The importance of studying iron uptake and homeostasis in pathogenic microorganisms is reflected by the fact that iron is an essential element for survival due to the requirement of hemoproteins for many important metabolic processes; hence, an iron transporter presents an attractive drug target. In *Schistosoma mansoni*, two isoforms of DMT1 have roles in iron transport, and immunofluorescence imaging revealed a surface-associated pathway for iron absorption, with both DMT1 isoforms mainly localizing to the tegument (Smyth et al., 2006). In *C. elegans,* an important model for the study of parasitic nematodes, SMF-1 and SMF-2 have 55–58% amino acid identity to mammalian DMT1. SMF-1 is highly expressed in the apical intestinal membrane; SMF-2 is mainly cytoplasmic and is also expressed in the pharyngeal epithelium (Bandyopadhyay et al., 2009).

We expressed a cDNA sequence encoding BmDMT1 in an iron transport-deficient yeast strain that has null mutations in the genes encoding the low and high-iron affinity transporters Fet3 and Fet4. Expression of BmDMT1 rescued growth of this strain in low-iron medium, which strongly suggests that this protein is a functional iron transporter.

We detected expression of BmDMT1 in microfilariae, L3, L4, adult females and adult males by qPCR; BmDMT1 mRNA was most abundant in adult females, with lowest levels in adult males relative to microfilariae. Higher expression of BmDMT1 in adult females than in other life stages is consistent with the high requirement of iron needed for the synthesis of hemoproteins essential for the formation of developing embryos and uterine microfilariae (which also require heme production by *Wolbachia*).

In adult female *B. malayi* and *O. volvulus*, the high level of BmDMT1 expression in developing eggs in the ovaries suggests a high demand for iron in these tissues, consistent with a requirement for hemoprotein synthesis in all new cells and tissues. We consistently detected staining in the intestinal lining, suggesting that this is the primary site for iron absorption. Recently published mass spectrometry data from a proteomic analysis of the body wall, digestive tract, and reproductive tissue of *B. malayi* confirmed the presence of BmDMT1 in the intestine and reproductive compartments, supporting our results (Morris et al., 2015). Gene-set enrichment analysis revealed an abundance of digestive tract proteins, such as proteolytic enzymes and transporters, suggesting that the intestine may be involved in both digestion and absorption of nutrients. Howells and Chen showed significant transcuticular uptake of d-glucose, l-leucine, and adenosine by adult *B. pahangi in vitro* and, although they were unable to detect oral uptake *in vitro,* demonstrated ingestion of trypan blue *in vivo* (Howells and Chen, 1981). The general belief has been that filariae obtain nutrients primarily across the cuticle. However, as free iron levels are extremely low in host biofluids, with iron tightly bound to transferrin, lactoferrin or hemoproteins such as hemoglobin, oral ingestion of these sources of iron would be required by filariids. The presence of DMT1 in the intestinal brush border of *B. malayi* and *O. volvulus* provides support for the hypothesis that oral ingestion of heme or iron-containing proteins is necessary for the survival of these parasites in the host.

*Brugia* spp. and most other filariae harbour an obligate endosymbiont bacteria which is crucial for development and reproduction. Tissue and stage-specific distribution of *Wolbachia* has been shown using a monoclonal antibody directed against *Wolbachia* surface protein and *in situ* hybridization targeting *Wolbachia* 16S rRNA (Fischer et al., 2011). In inseminated adult females, *Wolbachia* was present in the ovaries, embryos and in decreasing numbers in the lateral chords. In adult males, *Wolbachia* was present in the testes and lateral chords near testicular tissue, but was not observed in mature spermatids or spermatozoa. The presence of DMT1 in these tissues in *B. malayi* and *O. volvulus* suggests the high requirement of iron possibly for the biosynthesis of heme. *Brugia* spp. are incapable of synthesizing heme *de novo* and require *Wolbachia* for this purpose. *Wolbachia* contains all but one heme biosynthetic gene (protoporphyrinogen-IX oxidase), which is also not present in many heme-producing bacteria (Wu et al., 2009). Ferrochelatase (FeCH), which is involved in the last step of heme biosynthesis, is found in both the *Wolbachia* and *Brugia* genomes. Interestingly, *in situ* hybridization to localize FeCH expression in adult *B. malayi* females and males (Wu et al., 2013) shows an expression pattern very similar to what we observed for DMT1. The importance of the heme biosynthetic pathway for parasite survival has been demonstrated and is an attractive pathway for drug target (Slatko et al., 2010; Strubing et al., 2010; Wu et al., 2009). This leads us to conclude that BmDMT1 could also be a legitimate drug target, since inhibition of DMT1 would deprive the parasite of iron, a trace element crucial for the biosynthesis of heme by *Wolbachia* and consequently long-term parasite survival and reproduction. This conclusion also supports the hypothesis that the prolonged sterilization of adult filariae following exposure to ivermectin, as well as to anti-*Wolbachia* antibiotics, may arise from iron/heme deprivation due to the inability to obtain iron and then heme following ivermectin-induced pharyngeal paralysis or depletion of *Wolbachia* (Garrick et al., 2003), providing a common pathway for the effects of antibiotics and ivermectin on this process.

## 5 Conclusions

Our results support the hypothesis that BmDMT1 is an iron transporter. The high expression of this gene observed in the intestine indicates that this tissue is a primary iron absorption site for adult filariae, and the intense staining in the lateral cords and reproductive tissue of both males and females suggests that there is a high need for iron in these tissues. Interestingly, these are also tissues inhabited by *Wolbachia*, consistent with the need for iron for the biosynthesis of heme by this endosymbiont.

### Financial support

This work was supported by Natural Sciences and Engineering Research Council (NSERC) (2016-06602) of Canada, the Canada Research Chairs (950-228045) and the FRQNT Center for Host-Parasite Interactions.

## Conflicts of interest

The authors declare no conflicts of interest.
